# Selection of best videos of the year for 2020

**DOI:** 10.1590/S1677-5538.IBJU.2021.01.03

**Published:** 2020-11-18

**Affiliations:** 1 Moffitt Cancer Center Department of GU Oncology FL USA Department of GU Oncology Moffitt Cancer Center, FL, USA; 2 Moffitt Cancer Center Department of Tumor Biology FL USA Department of Tumor Biology Moffitt Cancer Center, FL, USA

## COMMENT

Esteemed readers and colleagues, I hope this message finds you and your families well in this challenging time of an ongoing global pandemic that has affected us all. I know this past year has brought about challenges beyond any of us could foresee but knowing the dedication and resilience of all of you, I know this is a temporary hurdle that all of us will not only overcome but will make us stronger and united. This past year has been another exceptional year for the International Brazilian Journal of Urology with the publication of a number of impactful scientific manuscripts and videos under the leadership of our new editor-in-chief Dr. Luciano A Favorito who instills great vision and foresight to our endearing journal for the many years to come. As is customary every year since taking on in my role as editor of the video section, I am pleased to announce the selection for best videos of the year for 2020. Many stringent criteria are used in making this selection including originality, innovation, and its inherent ability to optimize and positively impact patient care. Making this selection is truthfully never easy as we receive some truly outstanding videos every year and are privileged that international highly regarded colleagues specifically target our journal as their preferred journal for submission.

On that note, I am pleased to announce the selection of the 1st prize for best video of the year to “Beyond traditional frontiers: robotic total pelvic exenteration” by Dr. Tamhankar and colleagues from the TATA Memorial Hospital in Mumbai, India published in the November-December 2020 (1). This video is absolutely superb in its depiction on how a robotic minimally invasive platform can be used to resect a large soft tissue sarcoma of the pelvis (over 13 cm in diameter) and doing so while strictly adhering to the oncologic principles of complete tumor eradication with negative surgical margins. The surgery procedure was completed within an appropriate surgical timeline and favorable perioperative outcomes including acceptable blood loss as well as excellent patient post-operative outcomes. As highlighted by the authors, a critical element to successfully completing these complex surgeries is the engagement of a dedicated and coordinated multidisciplinary team. I highly encourage colleagues considering to complete such surgical procedures using a minimally invasive approach to refer to this video and its detailing of technical points and refinements.

The selection for 2nd prize for best video of the year in 2020 is “Analysis of surgeon biometrics during open and robotic radical cystectomy with electromyography and motion capture analysis” by Dr. Baumgarten and colleagues from the University of South Florida departments of urology and physical therapy with this work being published in the January-February 2020 (2). This video is incredibly innovative in concept and completion highlighting through kinematic and electromyography analyses of a single surgeon completing both open and robotic radical cystectomy, the large muscle groups engaged and potentially impacted in completing such elaborate and complex urological surgical procedures. There is a paucity of literature on the subject matter which is vital in optimizing surgical ergonomics and career sustainability for surgeons. We often set the benchmark of any successful surgical procedure patient related outcomes but of critical importance is as well the impact of completing these operations has on the surgeon as is often depicted in the phrase “we need to take care of ourselves before taking care of others.”

Lastly but certainly not least, the selection for 3rd prize for best video of the year is awarded to “Single port robot-assisted transperitoneal kidney transplant using the SP surgical system in a pre-clinical model” by Dr. Garisto and colleagues in the department of urology at the Cleveland Clinic in Ohio. This work was published in the July-August 2020 (3). This video provides an excellent depiction on how single port robotic surgery can be used to perform highly complex transplant surgery potentially in multiple abdominal/pelvic quadrants without the need for re-docking. This group of investigators led by Dr. Jihad Kaouk has been early adopters and innovators on the use of this single port minimally invasive surgical platform to conduct in a very methodical approach increasingly complex surgical procedures. Of note, in this instance they have refined the surgical approach using a pre-clinical model prior to its integration in their therapeutic paradigm most notably for transplant surgery where the bar of acceptable outcomes is set very high for transplant graft viability particularly as the numbers of transplant donors remains a very limited and finite resource in most healthcare systems and countries.

In conclusion, I would like to thank all of our readers for your strong support towards our journal and its video section, our success and recognition would not be possible without all of you. I strongly encourage international colleagues having interesting/innovative videos to consider submitting their work to our journal as we are continually seeking to highlight new approaches or surgical concepts as a means of offering our patients ultimately the most effective and minimally morbid surgical options in caring for their conditions.

## References

[1] 1. Tamhankar AS, Chaturvedi H, Gautam G. Beyond traditional frontiers: robotic total pelvic exenteration. Int Braz J Urol. 2020;46:1112. 10.1590/S1677-5538.IBJU.2019.0302 PMC7527094 32822147

[2] 2. Baumgarten A, Kim JK, Robison J, Mayer J, Hardwick D, Patel T. Analysis of surgeon biometrics during open and robotic radical cystectomy with electromyography and motion capture analysis. Int Braz J Urol. 2020;46:138. 10.1590/S1677-5538.IBJU.2019.0163 PMC6968904 31408292

[3] 3. Garisto J, Eltemamy M, Bertolo R, Miller E, Wee A, Kaouk J. Single port robot-assisted transperitoneal kidney transplant using the SP® surgical system in a pre-clinical model. Int Braz J Urol. 2020;46:680-1. 10.1590/S1677-5538.IBJU.2019.191 PMC7239304 32374143

